# Bidirectional Mendelian randomization analysis investigating the genetic association between primary breast cancer and colorectal cancer

**DOI:** 10.3389/fimmu.2023.1260941

**Published:** 2024-01-12

**Authors:** Yi Liu, Mingxuan Si, Yawei Qian, Yang Liu, Zichen Wang, Tongyu Zhang, Zhenhuan Wang, Kun Ye, Cuijuan Xiang, Linlin Xu, Yanping Zhang, Zhihan Xiao

**Affiliations:** ^1^ Department of Digestive System, Anqing Municipal Hospital, Anqing, China; ^2^ Department of Thoracic Surgery, Renji Hospital, Shanghai Jiao Tong University School of Medicine, Shanghai, China; ^3^ Department of General Surgery, The First Affiliated Hospital of Nanjing Medical University, Nanjing, China; ^4^ School of Economics and Management, Wuhan University, Wuhan, China; ^5^ Department of Thoracic Surgery, The First Affiliated Hospital of Nanjing Medical University, Nanjing, China; ^6^ Department of Cardiothoracic Surgery, Wuhu Second People’s Hospital, Wuhu, China

**Keywords:** colorectal cancer, breast cancer, GWAS, Mendelian randomization, GRS

## Abstract

**Purpose:**

With the advancement in early diagnosis and treatment, the prognosis for individuals diagnosed with breast cancer (BC) has improved significantly. The prognosis of primary breast cancer (PBC) survivors can be significantly influenced by the occurrence of colorectal cancer (CRC) as a secondary primary cancer (SPC). The objective of this study is to explore the possible genetic association between PBC and CRC, aiming to lay a groundwork for the development of preventive strategies against SPC-CRC following BC surgery.

**Methods:**

We employed a bidirectional two-sample Mendelian randomization (MR) approach to thoroughly examine genetic instrumental variables (IVs) derived from genome-wide association studies (GWAS) conducted on PBC and CRC. And applied inverse variance weighted (IVW) and multiple other MR methods (weighted median, simple median, MR-PRESSO and MR-RAPS) to evaluate the association between the two cancers (PBC and CRC) at genetic level. Furthermore, the robustness of the findings was further confirmed through the utilization of the genetic risk score (GRS) method in a secondary analysis.

**Results:**

Forward MR analysis, a total of 179 BC genetic IVs, 25 estrogen receptor-negative (ER-) genetic IVs and 135 ER-positive (ER+) genetic IVs were screened. Reverse MR analysis, 179 genetic IVs of CRC, 25 genetic IVs of colon cancer, 135 genetic IVs of rectal cancer, 25 genetic IVs of left colon cancer and 135 genetic IVs of right colon cancer were screened. IVW and other MR methods found no significant genetic association between PBC and CRC (*P* > 0.05). Subgroup analysis also showed that ER- BC and ER+ BC were not correlated with the occurrence of CRC (*P* > 0.05). The findings of the secondary analysis using GRS were consistent with those obtained from the primary analysis, thereby confirming the robustness and reliability of this study.

**Conclusions:**

Our findings do not provide any evidence supporting the association between PBC and CRC at the genetic level. Further large-scale prospective studies are warranted to replicate our findings.

## 1 Introduction

In 2020, breast cancer (BC) became the most prevalent form of cancer globally, with 226,419 new cases and 684,996 deaths. It ranked first in terms of both incidence and mortality (1). Fortunately, due to advancements in early detection and treatment, the 5-year survival rate for BC is about 89% as reported by the Surveillance, Epidemiology, and End Results (SEER) Program (2). However, as the survival time of BC patients prolongs, the incidence of second primary cancers (SPCs) increases, making BC a significant risk factor for SPC development (3–5). Based on the data provided by the American Cancer Society, a significant number of individuals in the United States have successfully overcome BC, emphasizing the importance of conducting screenings for secondary primary cancers (SPCs). Among these SPCs, it is crucial to prioritize regular examinations for prevalent types like colorectal cancer (CRC) to ensure the ongoing well-being of this extensive group of patients.

CRC impacts approximately 150,000 individuals annually within the United States and stands as the second most prevalent cause of cancer-related fatalities, resulting in the loss of 50,000 lives each year (6). Previous studies have indicated that BC survivors have a standardized incidence ratio (SIR) for CRC as high as 1.59. Moreover, individuals with BRCA mutations may experience a nearly five-fold increase in CRC risk. These findings indicate the necessity for earlier or more frequent CRC screening subsequent to BC surgery (7–10). Besides, existing observational studies suggest a possible link between BC and CRC, which is one of the primary forms of SPC. However, due to inherent limitations in such studies like confounding factors, the precise genetic-level connection between BC and CRC remains uncertain (11–16).

Mendelian randomization (MR) is a commonly employed method in genetic epidemiology for inferring causality (17). In recent years, as MR research methods have advanced, they have become a preferred approach for inferring genetic-level associations between two complex diseases, allowing for a better understanding of their pathogenesis. In 2021, Li et al. conducted a comprehensive investigation of the correlation between Parkinson’s disease and rheumatoid arthritis by performing two-sample MR analysis based on a large sample genome-wide association study (GWAS) (18). In 2023, Chen et al. employed two-sample MR to examine the association between Inflammatory bowel disease and prostate cancer. This study does not support a causal association of Inflammatory bowel disease and prostate cancer (19). In our research, our objective is to employ PBC and CRC GWAS data to investigate the genetic-level association between the two diseases using a two-sample MR analysis. This investigation will serve as a foundation for the development of clinical prevention strategies for subsequent CRC after BC.

## 2 Materials and methods

### 2.1 Sources of data

The instrumental variables (IVs) that associated with PBC were obtained from the largest GWAS conducted to date. This study, published by Kyriaki et al. in 2017, consisted of a large sample size, including 122,977 BC cases and 105,974 controls (20). Regarding the reverse analysis, we employed genetic IVs associated with CRC, which were derived from two recent meta-analyses of GWAS specifically focusing on CRC risk (21). The PBC and CRC GWAS summary statistics were obtained from GeneATLAS (http://geneatlas.roslin.ed.ac.uk/). Gene ATLAS is a comprehensive database that contains associations between hundreds of traits and millions of variants, which have been identified using the UK Biobank cohort. The study exclusively recruited participants of European ancestry.

### 2.2 Selection of IVs

The MR analysis aims to assess the impact of a predictor on an outcome. To ensure the validity of IVs, the fulfillment of three assumptions is necessary: (a) the independence of the IVs from the outcome should be taken into account when evaluating the exposure, which is restriction; (b) the correlation between the IVs and the exposure is necessary, which is commonly referred to as the “relevance” assumption; and (c) the independence of the IVs from any potential confounding factors, whether observed or unobserved, should be ensured (referred to as the assumption of “exchangeability”) (22, 23). Therefore, genetic IVs for overall BC, ER- BC, ER+ BC, overall CRC, colon cancer, rectal cancer, left CRC and right CRC were constructed according to the following criteria (24, 25): (a) LD, linkage disequilibrium among IVs is measured using the r^2^ statistic, with a threshold of less than 0.001 within a window size of 500 kb (Genetic variants in close genomic regions have a tendency to co-inherit, which is referred to as LD. When the presence of LD is observed alongside genetic variants, the information provided by each variant does not exist in isolation from one another. Consequently, when these genetic variants are interrelated as IVs, the estimation of effects can become biased); (b) *P* < 5 × 10^−8^ (In the GWAS study, this criterion demonstrated a significant correlation between single-nucleotide polymorphisms (SNPs) and the disease); (c) nonpalindromic SNPs (Palindromic sequences refer to DNA strands where the order of bases in SNPs is identical in both the forward and reverse directions. In situations where the gene responsible for the outcome effect has a low frequency, it becomes challenging to determine whether the sequence is in the forward or reverse orientation); (d) minor allele frequency (MAF) > 0.01 (The prevalence of mutations within the population is observed to be greater than 1%); (e) exclusion of IVs linked to confounding factors was performed through the utilization of PhenoScanner (In the process of conducting MR analysis, it is essential to address any potential confounding factors that may lead to associations between IVs and the outcome. This step is crucial in order to enhance the reliability and validity of research findings).

### 2.3 MR analyses

The primary analyses were performed utilizing the inverse variance weighted (IVW) method. The IVW approach, which is widely adopted and considered the predominant method for MR analysis, employs a meta-analysis method to combine ratio estimates of SNPs in an inverse variance weighted manner (26–28). The IVW methodology encompasses both the random-effects IVW and the fixed-effects IVW method. In cases where heterogeneity is observed in the MR analysis, we will utilize the random-effects IVW method, as it demonstrates reduced susceptibility to biases arising from weaker SNP-exposure associations (29). Furthermore, the simple median, weighted median, MR-RAPS, MR-PRESSO, and MR-Egger methods are employed to assess the genetic-level associations between BC and CRC. The simple median and weighted median approaches are utilized in this study, as they possess a high tolerance for pleiotropic IVs. The primary distinction between these two approaches pertains to the handling of estimated medians (The weighted median method incorporates distinct weights for each value, whereas the simple median method assigns uniform weightage to all values) (29, 30). The MR-RAPS approach, incorporating a Huber loss function, is capable of effectively capturing the random-effects distribution of pleiotropic effects. This approach is highly advised as a valuable technique for performing routine MR analysis, especially in scenarios involving intricate characteristics that encompass both the variables of exposure and outcome (31). The MR-PRESSO approach is employed in this study, assuming that a minimum of 50% of the genetic variants serve as valid IVs. This method takes into account both horizontal pleiotropy and the Instrument Strength Independent of Direct Effect (InSIDE) assumption. In addition to identifying genetic IVs that deviate from the norm, the MR-PRESSO technique provides revised estimates by eliminating these exceptional cases (32). The MR-Egger regression method involves conducting a linear regression analysis with weighted coefficients for the outcome and exposure variables, is capable of identifying certain deviations from the standard IVs assumptions. Furthermore, it offers a non-violation-prone estimation of the effect (33).

### 2.4 Genetic risk scores

In order to corroborate the aforementioned MR findings, a secondary analysis was conducted utilizing the GRS approach. The analyses were carried out employing R software (version 3.5.3) and the “gtx” R package (windows version 0.0.8). Specifically, the GRS function within the grs.summary module was utilized, which utilized summarized data from single SNP associations derived from GWAS results. This technique is akin to an additive GRS regression method (34). For uncorrelated SNPs, the causal estimate α value can be estimated by $\alpha \approx \frac{\sum^{}\text{ω}\beta se_{\beta }^{-2}}{{\;}_{\sum }^{{\text{ω}}^{2}}se_{\beta }^{-2}}$ , and the standard error se_α_ can be estimated by $se_{\alpha }\approx \frac{1}{{\;}_{\sum }^{\omega^{2}}se_{\beta }^{-2}}$ . In this context, ω represents the estimated effects on the intermediate trait or biomarker, while *β* values indicate the estimated effects on the response variable or outcome, accompanied by standard errors (se*_β_*) (34, 35).

### 2.5 Horizontal pleiotropy and heterogeneity test

To estimate pleiotropy, the MR-Egger regression technique was employed, while heterogeneity was assessed using Cochran’s Q test. To rule out the occurrence of horizontal pleiotropy, we verified that the *P* value of the MR-Egger intercept was above 0.05. If the *P* value of Cochran’s Q test was less than 0.05, we employed a multiplicative random-effects model for IVW as our final results; otherwise, a fixed-effects model was used (35). The F statistic is utilized to assess the strength of the association between the SNP and the exposures (36). If the F statistic is greater than 10, it indicates the absence of weak IVs.

A comprehensive statistical analysis was performed, and the level of statistical significance was established at *P* < 0.05. The analyses were conducted using R version 4.3.0 along with the utilization packages such as “MendelianRandomization”, “TwosampleMR”, “RAPS”, and “PRESSO” (37).

## 3 Results

### 3.1 MR analysis results of BC to CRC (forward MR)

#### 3.1.1 Screen and validation of IVs

**Figure 1:**
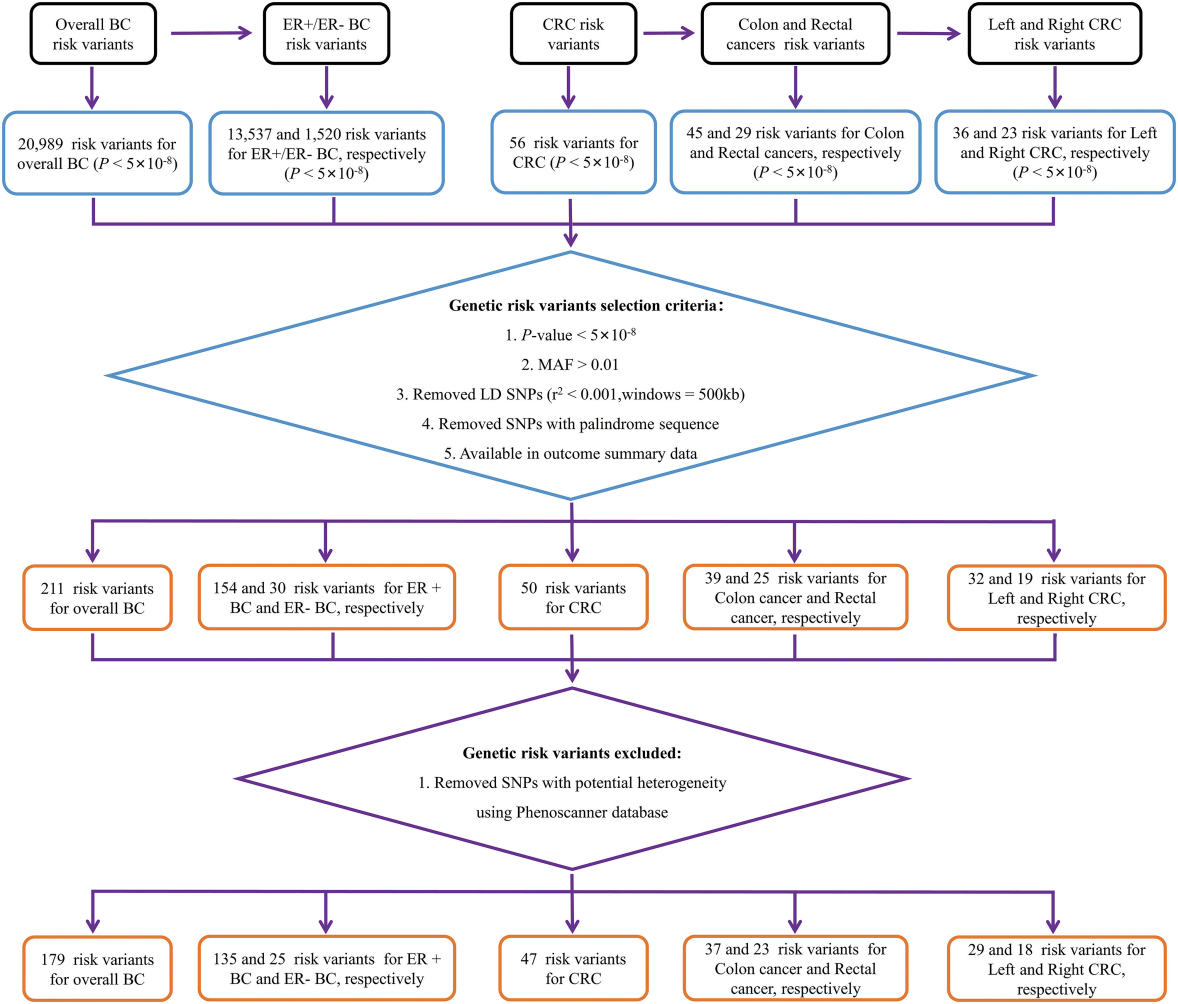
Flow chart of genetic variables screening. BC, breast cancer; CRC, colorectal cancer; MAF, minor allele frequency; ER-, estrogen receptor-negative; ER+, estrogen receptor-positive.

In BC to CRC MR analysis, 20,989 overall BC, 13,537 ER+ BC, and 1,520 ER- BC IVs reached significant differences in the GWAS study (*P* < 5×10^-8^). Following LD pruning and quality control measures (a: r^2^ measure of LD among IVs was found to be less than 0.001 within a 500-kb window; b: nonpalindromic single-nucleotide polymorphism; c: MAF > 0.01; c: Available in outcome summary data), 211 IVs were included as proxies of overall BC. 154 IVs were included as proxies of ER+ BC and 30 IVs were included as proxies of ER- BC. Then, we utilized the PhenoScanner database to eliminate potential confounding factors and successfully discovered 179 genetic IVs for overall BC, 135 IVs specifically associated with ER+ BC, and 25 IVs specifically linked to ER- BC. Details of genetic IVs selection were presented in **Figure 1** and basic characteristics along with summary effect estimates of included IVs on BC are presented in the **Supplementary Table S1**.

#### 3.1.2 Overall BC to CRC

**Figure 2:**
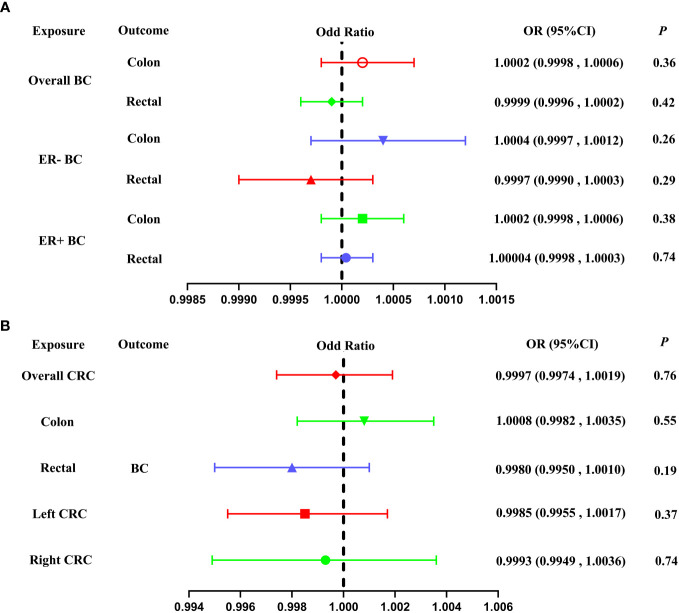
Forest plot of our Two-Sample Mendelian Randomization study based on the IVW method. **(A)** Mendelian randomization estimates of genetically predicted BC (overall BC, ER- BC and ER+ BC) on colon and rectal cancer risk (Forward MR analysis). **(B)** Mendelian randomization estimates of genetically predicted CRC (overall CRC, colon cancer, rectal cancer, left CRC and right CRC) on BC risk (Reverse MR analysis). BC, breast cancer; CRC, colorectal cancer; MAF, minor allele frequency; ER-, estrogen receptor-negative; ER+, estrogen receptor-positive; IVW, inverse variance weighted.

Our study used IVW as the primary analytical method to assess the relationship between BC and CRC. The IVW method provided no genetic relationship between overall BC and CRC (colon cancer: OR = 1.0002, 95% CI: 0.9998-1.0006, *P* = 0.36; rectal cancer: OR = 0.9999, 95% CI = 0.9996-1.0002, *P* = 0.42; **Table 1**; **Figure 2A**, **3A, B**). Similar results were observed by using the other different MR methods (weighted median, simple median, MR-RAPS and MR-PRESSO), indicating the lack of genetic association between overall BC and CRC (**Table 1**).

**Table 1:** Summarised results of Mendelian randomization study on BC to CRC.

		Colon cancer				Rectal cancer			
Group	MR approach	OR	95% CI		*P* value	OR	95% CI		*P* value
Overall BC	IVW (random)	1.0002	0.9998	1.0006	0.36	0.9999	0.9996	1.0002	0.42
	IVW (fix)	1.0002	0.9998	1.0006	0.36	0.9999	0.9996	1.0002	0.42
	Simple median	1.0001	0.9995	1.0008	0.72	0.9998	0.9993	1.0003	0.38
	Weighted median	1.0003	0.9996	1.0010	0.41	0.99997	0.9995	1.0004	0.89
	Penalized weighted median	1.0003	0.9996	1.0010	0.41	0.9999	0.9994	1.0004	0.68
	MR-RAPS	1.0002	0.9998	1.0006	0.36	0.9999	0.9996	1.0002	0.42
	MR-PRESSO (Raw)	1.0002	0.9998	1.0006	0.35	0.9998	0.9996	1.0002	0.41
	MR-PRESSO (Outlier-corrected)	NA	NA	NA	NA	NA	NA	NA	NA
	MR-Egger	1.0007	0.9997	1.0016	0.16	1.0003	0.9996	1.0009	0.40
Subgroup									
ER- BC	IVW (random)	1.0004	0.9997	1.0012	0.26	0.9997	0.9990	1.0003	0.29
	IVW (fix)	1.0004	0.9997	1.0012	0.26	0.9997	0.9991	1.0002	0.21
	Simple median	1.0003	0.9992	1.0014	0.59	0.9999	0.9991	1.0007	0.82
	Weighted median	1.0003	0.9992	1.0014	0.59	0.9995	0.9988	1.0003	0.22
	Penalized weighted median	1.0003	0.9992	1.0013	0.61	0.9995	0.9987	1.0003	0.19
	MR-RAPS	1.0004	0.9996	1.0012	0.26	0.9997	0.9992	1.0002	0.21
	MR-PRESSO (Raw)	1.0004	0.9997	1.0011	0.23	0.9997	0.9990	1.0003	0.3
	MR-PRESSO (Outlier-corrected)	NA	NA	NA	NA	NA	NA	NA	NA
	MR-Egger	0.9999	0.9978	1.0019	0.89	1.0001	0.9984	1.0018	0.90
ER+ BC	IVW (random)	1.0002	0.9998	1.0006	0.38	1.00004	0.9998	1.0003	0.74
	IVW (fix)	1.0002	0.9998	1.0006	0.38	1.00004	0.9998	1.0003	0.74
	Simple median	1.0002	0.9996	1.0009	0.50	1.0001	0.9997	1.0006	0.63
	Weighted median	1.0004	0.9997	1.0011	0.27	1.0002	0.9998	1.0007	0.33
	Penalized weighted median	1.0004	0.9997	1.0011	0.28	1.0002	0.9998	1.0006	0.36
	MR-RAPS	1.0002	0.9998	1.0006	0.38	1.00005	0.9998	1.0003	0.74
	MR-PRESSO (Raw)	1.0002	0.9998	1.0005	0.34	1.00004	0.9998	1.0003	0.73
	MR-PRESSO (Outlier-corrected)	NA	NA	NA	NA	NA	NA	NA	NA
	MR-Egger	1.0005	0.9997	1.0013	0.20	1.0004	0.9998	1.0009	0.20

NA, Not available.

**Figure 3:**
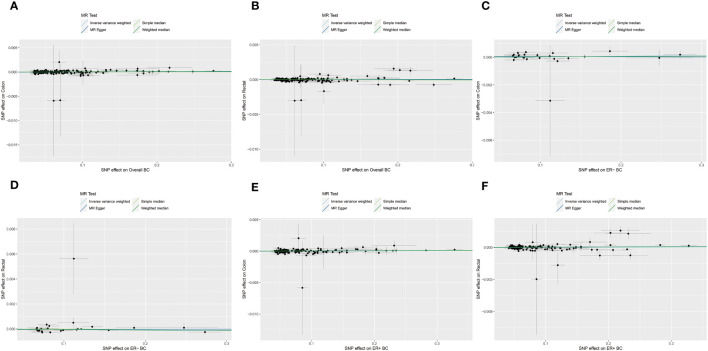
The scatterplots represents genetic instrument variables (IVs) association between BC and CRC (Forward MR analysis). **(A, B)** Plots of the effect size of each single nucleotide polymorphism (SNP) of overall BC on colon cancer **(A)** and rectal cancer **(B)** risk. **(C, D)** Plots of the effect size of each SNP of ER- BC on colon cancer **(C)** and rectal cancer **(D)** risk. **(E, F)** Plots of the effect size of each SNP of ER+ BC on colon cancer **(E)** and rectal cancer **(F)** risk. BC, breast cancer; CRC, colorectal cancer; ER-, estrogen receptor-negative; ER+, estrogen receptor-positive.

About stratified analysis, the IVW method indicate that no association was found between genetic predisposition to ER- BC and CRC (colon cancer: OR= 1.0004, 95% CI = 0.9997-1.0012, *P* = 0.26; rectal cancer: OR = 0.9997, 95% CI = 0.9990-1.0003, *P* = 0.29; **Table 1**; **Figure 2A**, **3C, D**). Similarly, no genetic relationship was found between ER+ BC and CRC (colon cancer: OR = 1.0002, 95% CI = 0.9998-1.0006, *P* = 0.38; rectal cancer: OR = 1.00004, 95%CI = 0.9998-1.0003, *P* = 0.74; **Table 1**; **Figure 2A**, **3E, F**). Similar results were observed by using the other different MR methods (weighted median, simple median, MR-RAPS and MR-PRESSO), indicating the lack of genetic association between ER- or ER+ BC and CRC (**Table 1**).

### 3.2 MR analysis results of CRC to BC (reverse MR)

#### 3.2.1 Screen and validation of IVs

To assess the effect of reverse MR analysis, 56 overall CRC, 45 colon cancer, 29 rectal cancer, 36 left CRC and 23 right CRC IVs reached significant differences in the GWAS study (5×10^-8^). After LD pruning and quality control measures (a: r^2^ measure of LD among IVs was found to be less than 0.001 within a 500-kb window; b: nonpalindromic single-nucleotide polymorphism; c: MAF > 0.01; c: Available in outcome summary data), 50 variants were included as proxies of overall CRC. 39, 25, 32 and 19 IVs were included as proxies of colon cancer, rectal cancer, left CRC and right CRC, respectively. Then, we utilized the PhenoScanner database to eliminate any potential confounding factors associated with IVs. Our analysis resulted in the identification of 47 genetic IVs for overall CRC, 37 IVs for colon cancer, 23 IVs for rectal cancer, and 29 and 18 IVs for left and right CRC respectively. Details of genetic IVs selection were presented in **Figure 1** and basic characteristics along with summary effect estimates of included IVs on CRC are presented in the **Supplementary Table S2**.

#### 3.2.2 Overall CRC to BC

**Figure 4:**
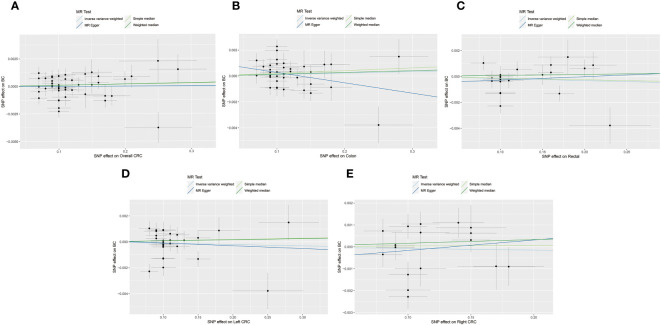
The scatterplots represents genetic instrument variables (IVs) association between CRC and BC (Reverse MR analysis). **(A)** Plots of the effect size of each single nucleotide polymorphism (SNP) of overall CRC on BC risk. **(B)** Plots of the effect size of each SNP of colon cancer on BC risk. **(C)** Plots of the effect size of each SNP of rectal cancer on BC risk. **(D)** Plots of the effect size of each SNP of left CRC on BC risk. **(E)** Plots of the effect size of each SNP of right CRC on BC risk. CRC, colorectal cancer; BC, breast cancer.

The IVW method did not reveal any significant association between a genetic predisposition to CRC and BC (overall CRC: OR = 0.9997, 95% CI = 0.9974-1.0019, *P* = 0.76; colon cancer: OR = 1.0008, 95% CI = 0.9982-1.0035, *P* = 0.55; rectal cancer: OR = 0.9980, 95% CI = 0.9950-1.0010, *P* = 0.19; left CRC: OR = 0.9985, 95% CI = 0.9955-1.0017, *P* = 0.37; right CRC: OR = 0.9993, 95% CI = 0.9949-1.0036, *P* = 0.74, **Table 2**; **Figure 2B**, **4A–E**).

**Table 2:** Summarised results of Mendelian randomization study on CRC to BC.

Group	MR approach	OR	95% CI		*P* value
Overall CRC	IVW (random)	0.9997	0.9974	1.0019	0.76
	IVW (fix)	0.9997	0.9980	1.0013	0.68
	Simple median	0.9998	0.9972	1.0025	0.91
	Weighted median	1.0009	0.9983	1.0035	0.51
	MR-RAPS	0.9996	0.9980	1.0013	0.66
	MR-PRESSO (Raw)	0.9997	0.9974	1.0019	0.76
	MR-PRESSO (Outlier-corrected)	1.0002	0.9981	1.0022	0.89
	MR-Egger	0.9998	0.9924	1.0073	0.96
Subgroup					
Colon cancer	IVW (random)	1.0008	0.9982	1.0035	0.55
	IVW (fix)	1.0008	0.9990	1.0027	0.39
	Simple median	1.0012	0.9982	1.0042	0.45
	Weighted median	1.0011	0.9982	1.0040	0.46
	MR-RAPS	1.0009	0.9990	1.0027	0.37
	MR-PRESSO (Raw)	1.0008	0.9982	1.0035	0.55
	MR-PRESSO (Outlier-corrected)	1.0002	0.9978	1.0026	0.88
	MR-Egger	0.9916	0.9820	1.0014	0.09
Rectal cancer	IVW (random)	0.9980	0.9950	1.0010	0.19
	IVW (fix)	0.9980	0.9958	1.0001	0.07
	Simple median	0.9987	0.9951	1.0022	0.46
	Weighted median	0.9991	0.9958	1.0024	0.58
	MR-RAPS	0.9979	0.9957	1.0001	0.06
	MR-PRESSO (Raw)	0.9980	0.9950	1.0010	0.21
	MR-PRESSO (Outlier-corrected)	0.9988	0.9962	1.0014	0.38
	MR-Egger	1.0025	0.9918	1.0134	0.65
Left CRC	IVW (random)	0.9985	0.9955	1.0017	0.37
	IVW (fix)	0.9986	0.9965	1.0007	0.18
	Simple median	0.9998	0.9966	1.0031	0.92
	Weighted median	0.9993	0.9961	1.0025	0.67
	MR-RAPS	0.9985	0.9964	1.0006	0.16
	MR-PRESSO (Raw)	0.9986	0.9955	1.0017	0.37
	MR-PRESSO (Outlier-corrected)	0.9992	0.9964	1.0020	0.58
	MR-Egger	0.9972	0.9846	1.0100	0.67
Right CRC	IVW (random)	0.9993	0.9949	1.0036	0.74
	IVW (fix)	0.9993	0.9966	1.0019	0.59
	Simple median	1.0004	0.9962	1.0045	0.87
	Weighted median	1.0015	0.9974	1.0057	0.47
	MR-RAPS	0.9992	0.9965	1.0019	0.56
	MR-PRESSO (Raw)	0.9993	0.9949	1.0036	0.74
	MR-PRESSO (Outlier-corrected)	1.0006	0.9969	1.0044	0.75
	MR-Egger	1.0048	0.9863	1.0236	0.62

### 3.3 GRS analysis results

#### 3.3.1 GRS_BC_ to CRC

To validate the results of the above MR analysis, we further conducted a secondary MR analysis using the GRS. Consistent with the MR results of overall BC, ER- BC and ER+ BC to colon cancer and rectal cancer, the GRS**_BC_** revealed no potential association between BC and colon cancer (overall BC: OR = 1.0002, 95% CI = 0.9998-1.0006, *P* = 0.36; ER- BC: OR = 0.9999, 95% CI = 0.9996-1.0002, *P* = 0.42; ER+ BC: OR = 0.9999, 95% CI = 0.9996-1.0002, *P* = 0.42, **Table 3**) or rectal cancer (overall BC: OR = 1.0002, 95% CI = 0.9998-1.0006, *P* = 0.36; ER- BC: OR = 0.9999, 95% CI = 0.9996-1.0002, *P* = 0.42; ER+ BC: OR = 0.9999, 95% CI = 0.9996-1.0002, *P* = 0.42, **Table 3**).

**Table 3:** The Effect of the GRS between BC and CRC.

Exposure	Outcome	OR	95% CI		*P*-value
Overall BC	Colon cancer	1.0002	0.9998	1.0006	0.36
	Rectal cancer	0.9999	0.9996	1.0002	0.42
ER- BC	Colon cancer	1.0004	0.9997	1.0012	0.26
	Rectal cancer	0.9997	0.9991	1.0002	0.21
ER+ BC	Colon cancer	1.0002	0.9998	1.0006	0.38
	Rectal cancer	1.00005	0.9998	1.0003	0.74
Overall CRC		0.9997	0.9980	1.0013	0.68
Colon cancer		1.0008	0.9990	1.0027	0.39
Rectal cancer	BC	0.9980	0.9958	1.0001	0.07
Left CRC		0.9986	0.9965	1.0007	0.18
Right CRC		0.9993	0.9966	1.0019	0.59

#### 3.3.2 GRS_CRC_ to BC

In the reverse-direction, in line with the aforementioned MR findings on overall CRC, colon cancer, rectal cancer, left CRC and right CRC to BC, the GRS**_CRC_** revealed no genetic association between CRC and BC (overall CRC: OR = 0.9997, 95% CI = 0.9980-1.0013, *P* = 0.68; colon cancer: OR = 0.9997, 95% CI = 0.9980-1.0013, *P* = 0.68; rectal cancer: OR = 0.9997, 95% CI = 0.9980-1.0013, *P* = 0.68; left CRC: OR = 0.9997, 95% CI = 0.9980-1.0013, *P* = 0.68; right CRC: OR = 0.9997, 95% CI = 0.9980-1.0013, *P* = 0.68, **Table 3**).

### 3.4 Horizontal pleiotropy and heterogeneity test

In the analysis of CRC to BC MR, Cochrane’s Q tests revealed some degree of heterogeneity among the CRC IVs (overall CRC: *P* = 0.003; colon cancer: *P* = 0.003; rectal cancer: *P* = 0.003; left CRC: *P* = 0.003; right CRC: *P* = 0.003, **Table 4**). The presence of heterogeneity was not observed in other MR analysis. The MR-Egger regression analysis indicated the absence of horizontal pleiotropy in both the BC to CRC forward MR analysis and the CRC to BC reverse MR analysis (**Table 4**).

**Table 4:** Heterogeneity and Horizontal pleiotropy analysis.

Exposure	Outcome	Cochran’s Q statistic	Cochran’s Q P	MR-Egger intercept P
Overall BC	Colon cancer	165.782	0.73	0.27
	Rectal cancer	172.925	0.59	0.18
ER- BC	Colon cancer	20.208	0.68	0.55
	Rectal cancer	33.353	0.10	0.58
ER+ BC	Colon cancer	112.997	0.91	0.33
	Rectal cancer	123.113	0.74	0.20
Overall CRC	BC	86.974	0.0002	0.96
Colon cancer		74.169	0.0002	0.06
Rectal cancer		43.866	0.0037	0.39
Left CRC		61.990	0.0002	0.83
Right CRC		45.841	0.0002	0.55

## 4 Discussion

SPC refers to the occurrence of a second primary cancer in an individual who has already been diagnosed with a primary cancer for some time. In recent years, with the progress of cancer prevention, diagnosis and treatment, a large number of early-stage cancer patients have received timely and effective treatment, and the survival period of patients after treatment has been significantly extended. In 2014, 14.5 million early-stage cancer patients in the United States have achieved long-term survival (38). Previous studies have demonstrated a significantly higher prevalence of SPC in the cancer population compared to the normal population, with an increasing trend observed over time. Moreover, it has been found that more than 19% of patients with a follow-up duration exceeding 20 years may experience SPC (38). The incidence of SPC in BC patients is approximately 5%, with a specific risk ratio of 1.59 for developing CRC compared to the general population (6). As the proportion of SPC following BC gradually increases, researchers have shown significant interest incidence, treatment, and prognosis of SPC. CRC, being one of the most prevalent and fatal types of SPC, investigating the association between PBC and CRC incidence can contribute to the identification of high-risk patients for timely screening. This would facilitate prompt implementation of effective treatments to improve rates of survival.

The cause of SPC is still unclear. Precious observational studies suggest that genetic factors, environmental factors and lifestyle habits may be related to the occurrence of SPC (39). Meanwhile, existing clinical studies have found an increased incidence of SPC-CRC after BC, these studies often overlook confounding factors, making it difficult to determine whether the relationship between BC and CRC is independent of these confounding factors. For example, previous studies have shown that factors such as smoking, alcohol consumption, and BMI increase the risk of BC and CRC occurrence (40–46). It is still a clinical issue to be explored whether there is a correlation between BC and CRC and what level of correlation exists. Hence, we conducted a thorough screening of confounding factors associated with both BC and CRC using the PhenoScanner database. After adjusting for these confounding factors, MR analysis was conducted to explore the genetic-level association between BC and CRC, while accounting for potential confounders. The present study ultimately demonstrating no significant evidence of a causal relationship at the genetic level.

According to the 2021 guidelines from the United States Preventive Services Task Force (USPSTF) (47), it is recommended that individuals between the ages of 50 and 75 undergo colorectal cancer (CRC) screening. Individuals who have a family history of CRC, as well as those who are obese, have a long history of smoking, or engage in heavy alcohol consumption, are advised to undergo regular screening. This is due to their increased susceptibility to developing CRC. Previously, our team of researchers successfully established a noteworthy genetic correlation between primary lung cancer and CRC through MR method (25). However, in this study, our research team did not find a statistically significant association between PBC and CRC using MR approach. As a result, individuals with a history of primary lung cancer but not breast cancer should undergo regular screening, which may include tests such as colonoscopy, digital rectal examination, and fecal occult blood test. It is imperative to closely monitor the incidence of SPC-CRC in order to promptly initiate early intervention and treatment measures.

This study utilizes the two-sample MR approach to investigate the possible relationship between PBC and CRC, which offers clear advantages compared to observational studies. In this study, we using the PhenoScanner database conducted a comprehensive examination of confounding factors associated with both BC and CRC. We also took steps to remove IVs that were associated to these confounding factors to minimize the potential influence of horizontal pleiotropy on the genetic IVs. Moreover, the MR-PRESSO and MR-Egger methods employed to conduct further assessments on the impact of pleiotropy in order to enhance the credibility of the findings (48, 49). In addition, we used Cochran’s Q method to test the heterogeneity of IVs. If there was no significant heterogeneity in Cochran’s Q test, unbiased association estimation was performed by IVW linear regression. If there is significant heterogeneity, the random effects IVW model is used for analysis to ensure the correctness of the analysis results (29, 50). Secondly, in addition to the application of IVW approach and various MR methods as analysis methods, GRS method is also used for secondary analysis. Thirdly, we used subgroup analysis for BC and CRC for the first time, and identified that BC and CRC were not significantly associated. Additionally, it is important to acknowledge certain limitations in our study. Firstly, it is challenging to completely eliminate the impact of potential pleiotropy in any Mendelian randomization (MR) study, which can introduce bias in the estimates of causal effects (48). Nevertheless, no evidence of pleiotropic effects was found in the MR-Egger regression analysis, and consistent findings were obtained in sensitivity analyses conducted with various robust models. Moreover, this study only focused on a specific population, and the generalizability of the findings to the entire population still needs to be confirmed. Additionally, GWAS have the potential to offer novel insights into the genetic factors implicated in the development of PBC-CRC. However, further investigations are required to elucidate the precise mechanisms underlying the pathophysiology for a more comprehensive understanding. Lastly, our conclusion lacks validation by wet laboratory experiments. Combining wet laboratory experiments or clinical data to support gene discovery will enhance the robustness of our research.

In conclusion, we do not find clear evidence that genetic correlation between PBC and CRC. In order to validate the accuracy of our findings, future research based on large-scale prospective studies will be necessary.

## Data availability statement

The original contributions presented in the study are included in the article/**Supplementary Material**. Further inquiries can be directed to the corresponding authors.

## Author contributions

YiL: Conceptualization, Writing – review & editing, Investigation, Writing – original draft. MS: Writing – original draft, Data curation. YQ: Writing – original draft, Methodology. YaL: Investigation, Writing – review & editing. ZCW: Writing – review & editing, Data curation. TZ: Writing – review & editing, Formal analysis. ZHW: Formal analysis, Writing – original draft. KY: Formal analysis, Writing – review & editing. CX: Formal analysis, Writing – review & editing. LX: Writing – review & editing, Validation. ZX: Writing – review & editing, Conceptualization, Writing – original draft. YZ: Conceptualization, Writing – review & editing.
